# A Clinical Lecture on Recent Advances in the Surgery of the Vermiform Appendix

**Published:** 1898-05-14

**Authors:** 


### Medical Progress and Hospital Clinics.

[*The Editor will be glad to receive offers of co-operation and contributions from members of the profession. All letters should be addressed to* The Editor, at the Office, 28 & 29, Southampton Street, Strand, London, W.C.]

### A CLINICAL LECTURE ON RECENT ADVANCES IN THE SURGERY OF THE VERMIFORM APPENDIX.

Reclus considers that the best explanation of the etiology of appendicitis is the "stagnation, or natural blind fistula" theory, by which he means that appendicular accidents are due to the stagnation of a virulent liquid in the appendix. This exacerbation of the virulence of morbific germs in a stagnating organic fluid is well known in urinary pathology, and particularly in the various fermentations which take place behind a stricture in the bladder, the ureters, and the pelvis of the kidney. Such are the conditions which have led him to compare the inflamed appendix to a natural blind fistula. Allen, however, concludes that appendicitis in most cases is a disease resulting from gradual processes having their origin in disturbances of fœtal development. He regards a bend in the appendix as the first step in its inflammation, the bend interfering with the escape of the contents of the appendix into the intestine, and so producing muscular hypertrophy, appendicular colic, and perforation. The reason for assuming that the bend is primary rather than secondary is that the organ is found bent in many cases in which there is no external evidence of inflammation. The cæcum originally occupies the right hypochondriac region, and from this it gradually descends into the right iliac fossa. Both cæcum and appendix have their own mesentery, and the process of descent causes a change in the relations of these organs to each other, and in certain cases a bend of the appendix takes place. If this bend is sufficient to make the emptying of the appendix impossible, conditions of obstruction gradually take place which terminate in appendicitis. Dieulafoy regards closure of the appendicular canal as the cause of appendicitis. This may be brought about by concretions, local infection, and tumefaction of the walls, &c., and the appendicular attack, whether benign, grave, or violent, only declares itself when the closed cavity is actually constituted.

In the differential diagnosis of appendicitis Fowler calls attention to the following: Frequency—males, 80 per cent.; females, 20 per cent. History of an attack is usually that of an acute onset. Pain is usually acute and radiating. In subacute and chronic appendicitis the pain may be dull and localised. Vomiting is exceedingly common in appendicitis. Tenderness in the majority of cases is located over the site of the appendix. Chill or rigor is of infrequent occurrence in appendicitis. Fever is present in appendicitis, though its grade does not indicate the severity of the attack. Muscular tension is almost invariably absent in adnexa lesions. Tumour is rarely present before the third day in appendicitis. It is rare that a tumour of appendical origin can be felt per vaginam. The course is usually an acute one, while lesions of the adnexa are usually subacute or chronic.

Dumontpallier does not admit the propriety of substituting the term appendicitis in all cases for the typhlitis and perityphlitis of older writers. The latter, however, more frequently get well with medical treatment, and are, therefore, not so easily demonstrable. Gaillard found that among a total of 7,213 cases of "typhlitis" surgical operation was necessary only in 473, that is to say, 6·6 per cent.; among the other 6,740 cases of typhlitis, treated by medical measures, the death-rate was only 8 8 per cent. Renvers says that of 2,000 cases observed in six years in the German army recovery was obtained without operation in 96 per cent.; and in a series of 115 cases observed by Revilliod there were only eight deaths.

Syms divides cases of appendicitis into: (*a*) Benign. I. Acute, primary—(1) simple catarrhal, with or without concretion; (2) parietal, involving all the coats; and (3) parietal, with local adhesive peritonitis. II. Chronic: (1) recurrent; and (2) relapsing, with concretion, stenosis, or foreign body. (*b*) Malignant—(1) acute suppurative, with local fibrino-purulent peritonitis, by extension or perforation; (2) acute suppurative, with progressive fibrino-purulent peritonitis, by extension or perforation; (3) subacute gangrenous with localised fibrino-purulent peritonitis; (4) suppurative, with retro-cæcal cellulitis; and (5) gangrenous, with retro-cæcal cellulitis. The "fulminating" forms he divides into: (1) Acute purulent, with perforation and general peritonitis or peritoneal sepsis; and (2) acute gangrenous, with perforation, general peritonitis, or peritoneal sepsis.

Carstens sums up his experience thus: (1) Every patient with appendicitis should be promptly subjected to operation when the diagnosis is made. (2) In all mild cases in which the patients have had two attacks they should be operated upon in the interval, because (*a*) when they have had two attacks they will have more, and (*b*) any future attack may be fatal. (3) Patients who have had only one mild attack can be watched, as it might be one of those cases which are without recurrence; but if a second attack develops, the patient should be operated upon. (4) The earlier in the attack the operation is performed the better results can be obtained, the only exception being those rare cases of large abscess which develop slowly. McCosh and Hawkes consider that operative interference is indicated when at the end of twenty-four hours the following conditions exist: (1) If the patient looks ill; if there is vomiting and tympanites, with a rapid pulse. (2) If the patient looks ill and there is vomiting, even though the pulse and temperature are each under 100. (3) If the patient looks ill and the pulse is over 110. (4) If there is rapid and feeble pulse, and extreme tenderness. (5) If the local pain and tenderness, at first localised, tend to become general, even though other grave symptoms may be absent. (6) If local pain and tenderness have continued for more than four weeks, or if without diminution for more than two weeks. Beaussenat summarises the result of numerous experiments as follows: (1) Appendicitis is almost always due to infection. (2) Infection may occur through the blood vessels and lymphatics, but is generally of intestinal origin. (3) The most frequent agent is the bacillus coli communis. (4) Before the agent can act the vitality of the mucosa must be modified, or the defensive phagocytosis weakened. (5) The infective folliculitis which characterises every appendicitis spread by the peri-follicular adenitis, suppurative or not, is constant. (6) The predisposing causes create the initial lesion, or favour infection by diminishing the chances of resistance. (7) The predisposing cause, *par excellence*, is entero-colitis.

Talamon says that of 30 cases of plastic appendicitis, one was operated upon for intestinal obstruction on the sixth day; the appendix was resected in another on the twentieth for a recrudescence; the rest were treated medically, and all recovered. Before the second week one can hardly tell plastic cases from suppurating peritonitis. Armstrong reports 517 cases seen in the leading Montreal hospitals. In the total number of cases the average mortality was 12·8 per cent.; but from 1853 to 1890—that is, in pre-operative days—the mortality was 23·8 per cent. Of the 517 cases, 389 were operated on, and 128 treated without operation. In this latter list the mortality was 3·12. Of 319 cases, 81 were interval cases, in which there was not one death. Of 305 operated on in the acute stage 63 died. The author's rule was to advise operation at the end of 24 or 36 hours, if the patient was not improving.

Kammerer proposes a modified incision in quiescent appendicitis: An incision is made through the skin, subcutaneous tissues, and outer layer of the sheath of the rectus. The outer border of the muscle is exposed, and drawn towards the median line by retractors. The epigastric vessels are divided, if necessary, and an incision is made an inch from the semilunar line, through the posterior layer of the sheath, the fascia transversalis and peritoneum. After removal of the appendix this last incision is closed with catgut, the rectus muscle allowed to fall back in place, and the anterior layer of the sheath closed with catgut sutures, some of which pass through the muscle itself. The skin is finally sutured with catgut. A similar incision is described by Battle. Tuberculous disease of the abdomen occasionally simulates appendicitis, and Page, after referring to a case of Lockwood, quotes one of tuberculous ulceration of the cæcum upon which he operated on the supposition that it was a case of appendicitis. Bonoin reports a case of actinomycotic typhlitis, similar to those of Lang and Roux. Operation was resorted to, but after the formation of abscesses over the right ischial tuberosity and elsewhere, the patient died of hectic.

